# Improved field emission performance of carbon nanotube by introducing copper metallic particles

**DOI:** 10.1186/1556-276X-6-537

**Published:** 2011-10-03

**Authors:** Yiren Chen, Hong Jiang, Dabing Li, Hang Song, Zhiming Li, Xiaojuan Sun, Guoqing Miao, Haifeng Zhao

**Affiliations:** 1 Key Laboratory of Excited State Processes, Changchun Institute of Optics, Fine Mechanics and Physics, Chinese Academy of Sciences, 3888 Dongnanhu Road, Changchun, 130033, People's Republic of China; 2 Graduate School of the Chinese Academy of Sciences, Beijing 100039, People's Republic of China

## Abstract

To improve the field emission performance of carbon nanotubes (CNTs), a simple and low-cost method was adopted in this article. We introduced copper particles for decorating the CNTs so as to form copper particle-CNT composites. The composites were fabricated by electrophoretic deposition technique which produced copper metallic particles localized on the outer wall of CNTs and deposited them onto indium tin oxide (ITO) electrode. The results showed that the conductivity increased from 10^-5 ^to 4 × 10^-5 ^S while the turn-on field was reduced from 3.4 to 2.2 V/μm. Moreover, the field emission current tended to be undiminished after continuous emission for 24 h. The reasons were summarized that introducing copper metallic particles to decorate CNTs could increase the surface roughness of the CNTs which was beneficial to field emission, restrain field emission current from saturating when the applied electric field was above the critical field. In addition, it could also improve the electrical contact by increasing the contact area between CNT and ITO electrode that was beneficial to the electron transport and avoided instable electron emission caused by thermal injury of CNTs.

## Introduction

Carbon nanotubes (CNTs) have extensively been investigated since they were discovered by Iijima [1]. The earliest research on CNTs as field emitter was conducted by de Heer et al. [2], which lifted the curtain on the field emission application of CNTs. Followed by a large number of studies on CNTs, the characters of high aspect ratio, small radius of curvature, good electric conductivity, and excellent chemical stability have contributed to the superior field emission behaviors such as lower turn-on voltage and larger emitting current density which have been considered as excellent and potential field emission electron sources used in field emission display (FED) [3-8].

Although CNTs have several advantages mentioned above, as the key component of FED, they must be deposited on a substrate (such as indium tin oxide (ITO) conductive glass) for their applications, low-cost, large-scale area, and homogeneous deposition become the primary targets of CNTs-based field emitters on the display panel [9]. To meet these requirements, the electrophoretic deposition (EPD) technique was adopted. However, the problem of weak electrical contact has to be taken into account. As field emission electron sources, CNTs must have good adhesion and electric conductivity to the electrode so that they can exhibit excellent field emission performance, especially the stability of electron emission. Wang et al. [10] ascribed the instability in emission current to the structural damage during emission. Bonard et al. [11] attributed the failure of a CNT to the resistive heating at the contact to substrate. More investigations had revealed that emitting CNT would involve a self-heating process [12,13], which might result in subliming and melting of a CNT and ultimately caused a failure in field emission. Xu and colleagues [14] addressed the physical mechanism responsible for the breakdown process because of the self-heating of CNTs. To sum up, how to avoid the instability of electron emission because of thermal injury of bad contact to substrate is an urgent issue to be overcome. It was reported that the presence of a charging agent could improve the adhesion of CNTs to substrate in the EPD process [15]. Talin et al. [16] developed a method of EPD process with Mg (NO_3_)_2 _· 6H_2_O additive to precoat CNTs with Mg (OH)_2_, and then transformed the precoat into MgO by heat-treating that improved the adhesion of the CNTs. Above-mentioned method did not improve the electrical contact between the electrode and CNTs by reason that MgO was the dielectric material. It was also reported that nanostructured metal-CNT composites had a combination of high strength and good plasticity [17]. In this article, a simple and low-cost way to improve field emission performance especially stability of carbon nanotube field emission display (CNT-FED) using copper metallic particle-carbon nanotube (Cu-CNT) assemblies through EPD technique is investigated.

## Experimental method

**Figure 1:**
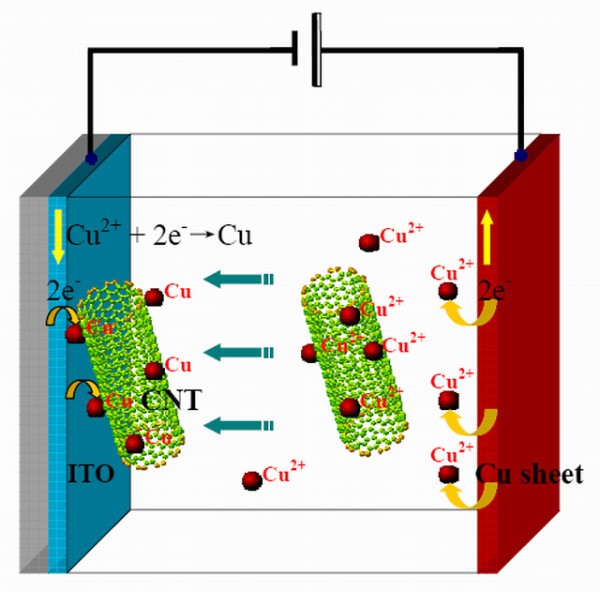
**Schematic of Cu-CNT assemblies' deposition by the EPD process**.

The fabrication of Cu-CNT assembly cathode is carried out by EPD process as shown schematically in Figure 1. First, the electrophoresis solution is prepared, using analytically pure isopropyl alcohol (IPA) as the solvent with Cu (NO_3_)_2 _· 3H_2_O additive. After evenly stirring, the initial concentration of Cu (NO_3_)_2 _· 3H_2_O in IPA is up to 5 × 10^-4 ^mg/L. In succession, CNTs will be added to the solution with a uniform dispersion by ultrasonic method. Second, an ITO conductive glass and a thin Cu sheet are placed parallel with a pitch of 5 cm in the electrophoretic liquid. ITO conductive glass connects to the negative electrode of DC voltage while Cu sheet to the positive one, when 50 V DC applied to them, the CNTs attached to Cu^2+ ^ions will be driven to move to the negative electrode under the electric field. When CNTs arrive at the ITO electrode, Cu^2+ ^ions adsorbed on the CNTs are reduced to form metallic Cu particles by reason of high electrical conductivity of CNT which allows electron to conduct from the Cu sheet to the outer layer of a CNT. Meanwhile, the atoms of Cu sheet lose 2e^- ^to form Cu^2+ ^ions and release into the IPA. This process can be described as:

(1) $${\text{Cu}}^{2+}+2{\text{e}}^{-}\underset{}{↔}\text{Cu}$$

Soon after finishing the deposition, the Cu-CNT film on ITO is rinsed in IPA and dried at 80°C for 10 min in the atmosphere, and then heated at 450°C in nitrogen atmosphere for half an hour to remove the residual solvents.

For the purpose of characterizing and analyzing, many kinds of test tools are adopted. The surface morphologies of samples are characterized using scanning electron microscope (SEM, Hitachi S-4800). To qualitatively analyze the presence of Cu element in Cu-CNT assembly cathode, the energy dispersive X-ray spectrum is measured by energy dispersive X-ray spectroscopy (EDS; Genesis 2000, EDAX, Inc.). The content of electrodeposited copper particles is identified using X-ray diffraction (XRD, Bruker D8 Focus, Bruker AXS, Inc.). For discussing the dispersion stability of suspension, zeta potential of the CNTs in electrophoresis solution is measured using Zetasizer 3000 (Malvern, Inc.). To evaluate the performance of CNTs by introducing Cu metallic particles, the field emission characteristic curve and electron emission stability curve are acquired by two Agilent 34401A sourcemeters (Agilent Technologies, Inc.) with one as ammeter and the other as voltmeter.

## Results and discussion

The focus points of the experiment mentioned above can be described as follows: the adsorption of Cu^2+ ^ions on the CNTs and the symmetrical dispersion of CNTs in suspension. The mechanism by which the Cu^2+ ^ions are sorbed onto CNTs can be attributable to electrostatic attraction and chemical interaction between the Cu^2+ ^ions and the surface functional groups of CNTs [18,19]. On the surface of CNTs, it generally exists the defects such as pentagons and heptagons which can introduce a quantity of oxygen-containing functional groups like carboxyl (-COOH) and hydroxyl (-OH) in IPA solution [20]. These functional groups, on the one hand, cause a rise in negative charge on surface of CNTs and absorb the Cu^2+ ^ions by electrostatic attraction, on the other hand, supply protons of carboxyl and hydroxyl to exchange with the Cu^2+ ^ions in solution.

Owing to the absorption of Cu^2+ ^ions on CNTs in the IPA solution, the zeta potential, with a concentration of Cu (NO_3_)_2 _· 3H_2_O up to 5 × 10^-4 ^mg/L, reaches +38.6 mV, meaning that the significantly electrostatic repulsion force induced by positive surface charges of CNTs is sufficient to prevent the agglomeration of CNTs in IPA solution which benefits to deposit.

**Figure 2:**
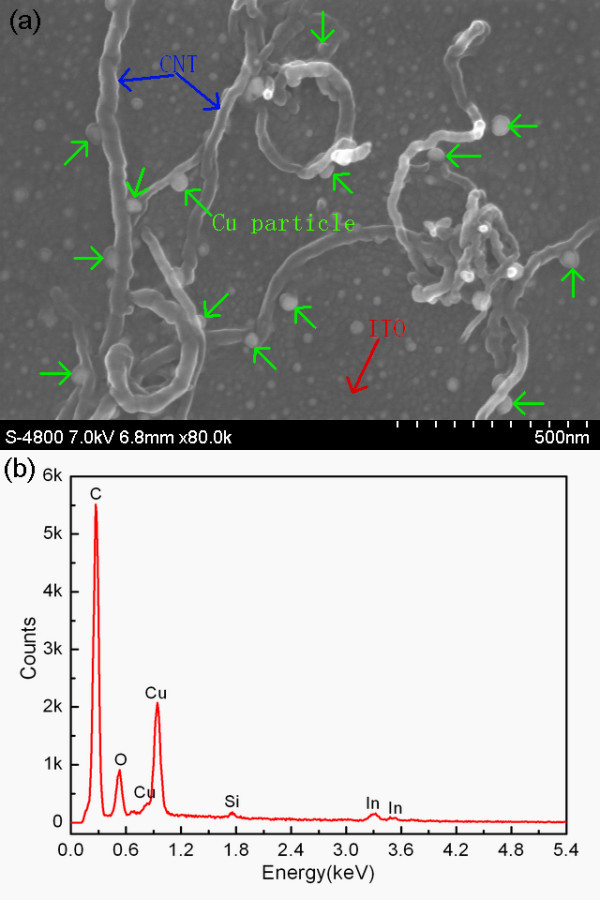
**SEM image and EDS of Cu-CNT assemblies**: **(a) **The morphology of Cu particles located between CNT and ITO electrode and **(b) **EDS of Cu-CNT assembly cathode.

**Figure 3:**
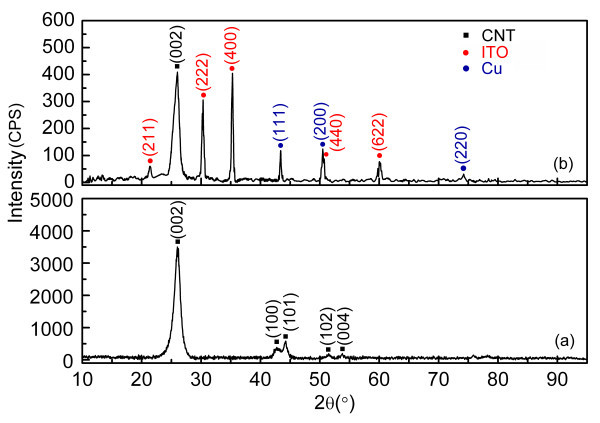
**XRD patterns**: **(a) **bare-CNTs; **(b) **Cu-CNT assemblies deposited on ITO conductive glass.

**Figure 4:**
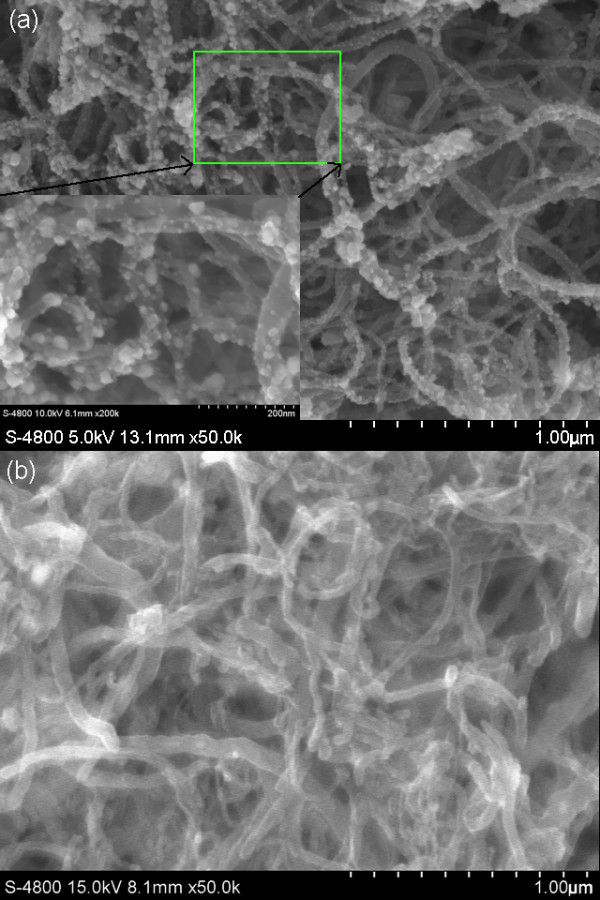
**Surface morphology of the CNT film on ITO electrode by means of the EPD process**: **(a) **Cu-CNT assemblies; **(b) **bare-CNTs.

A SEM image of Cu-CNT assemblies located on ITO electrode is presented in Figure 2a. The Cu particles (individually indicated by arrows) connect the CNTs to the ITO electrode which improves the contact area and increases the surface roughness of the CNT. To prove that the particles coated on surface of CNTs are Cu particles only, further investigations have been carried out. EDS of CNTs cathode is measured, as shown in Figure 2b, which qualitatively analyzes the presence of Cu element in the sample besides the elements of carbon and indium. In addition, Figure 3 gives the XRD patterns of (a) bare-CNTs and (b) Cu-CNT assemblies deposited on ITO electrode. In the diffraction pattern of Figure 3a, the dominant peak at 2θ = 26.02°and several weak peaks are observed, which correspond to the planes of (002), (100), (101), (102), and (004) of graphitized CNTs, respectively. As seen from Figure 3b, three groups of peaks are detected in the sample of Cu-CNT assemblies deposited on ITO electrode. Among them, one group contains three diffraction peaks at 2θ = 43.36°, 50.50°, and 74.23°with *hkl *reflections (111), (200), and (220), respectively, corresponding to the face-centric-cubic phase of Cu metal, another group shows five diffraction peaks at 21.46°, 30.36°, 35.30°, 50.62°, and 60.24°, corresponding to the planes of (211), (222), (400), (440), and (622) of ITO, other than the dominant diffraction peak of graphitized CNTs. The XRD patterns further demonstrate the presence of Cu metallic particles. To reflect the distinction of Cu-CNT assembly cathode intuitively, the surface morphology of the Cu-CNT assembly cathode and the bare-CNTs cathode are measured by FE-SEM, as shown in Figure 4a,b, respectively. In Figure 4a, it is apparent to see from partial enlarged detail that the surface of CNTs is coated with particles, which differs from the surface of CNTs in Figure 4b. The coated Cu particle size in Figure 4a, is about 10-50 nm. The density and size of the Cu particles could be controlled by the deposition time, electrical potential, and concentration of electrophoresis solution.

**Figure 5:**
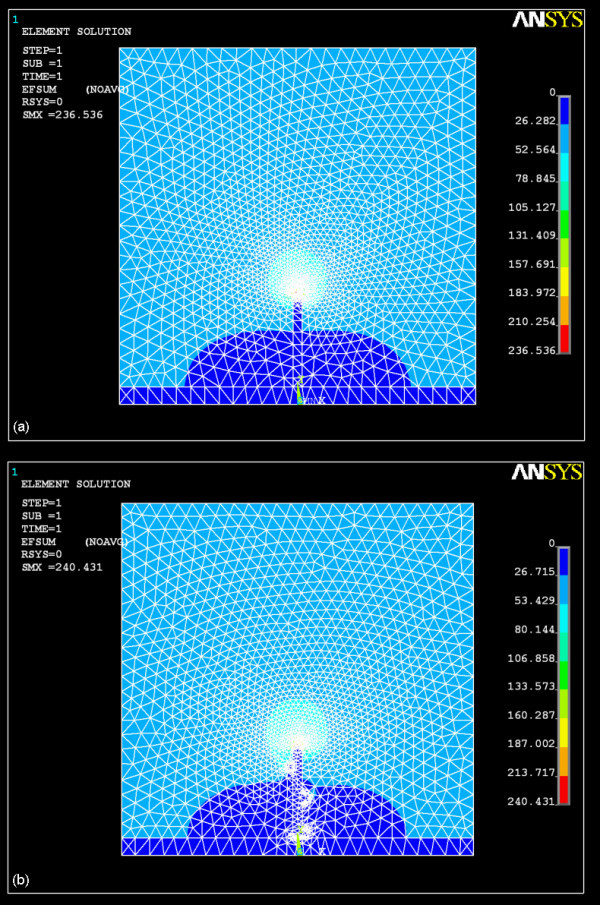
**The simulation of electric field distribution around CNT**: **(a) **bare-CNT; **(b) **Cu-CNT assembly.

As mentioned above, the presence of Cu particles on surface of CNT increases their surface roughness, which will be beneficial to field emission. To get a further illustration, we model a CNT as a one-dimensional object and simulate the electric field distribution around CNT with and without Cu particles on it. The electric field distribution can be seen in Figure 5. In contrast with Figure 5a,b, the participation of Cu particles in Figure 5b not only enhances the electric field distribution around CNT, but also introduces new field emitters.

**Figure 6:**
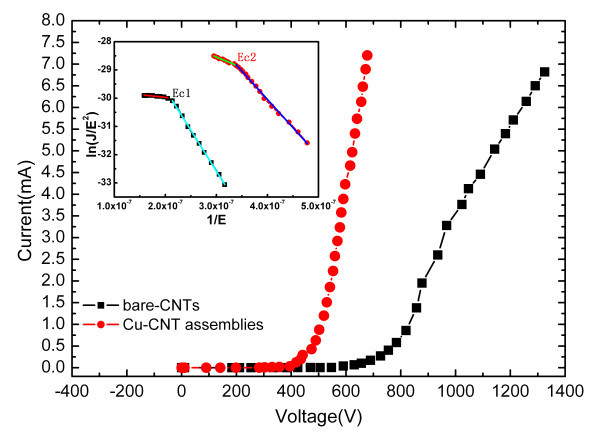
**Field emission curves and FN plots (inset)**.

The field emission characteristic curve and electron emission stability curve of both Cu-CNT assembly cathode and bare-CNTs cathode are measured in a vacuum chamber at a pressure of 9 × 10^-5 ^Pa, where the distance between cathode and anode plates is 200 μm. According to the results shown in Figure 6, the current-voltage performances of both cathodes are investigated. The inset of Figure 6 is illustrated by Fowler-Nordheim (FN) coordinates. We can obtain the turn-on field decreases from 3.4 to 2.2 V/μm. Both of the FN plots show a nonlinear characteristic and exhibit a critical field (signed as *E*_c_). The FN plots deviate from the original line when the applied electric field is above the critical field. When it is below the critical field (*E*_c_), both of the field emission processes follow the FN law. In contrast, the deviation from original line of Cu-CNT assemblies' one tends to be relatively gentle. In addition, although both of the FN plots have deviation when the applied electric field is above *E*_c_, the Cu-CNT assemblies' one tends to saturate slower than bare-CNT's. The reason for saturation is that a weak electrical contact between CNT and ITO electrode results in high resistance of interface so that the electron transport is restricted, and therefore, induce the current saturation at high applied electric field [11,21,22]. At the meantime, we also find that the contact conductivity increases from 10^-5 ^to 4 × 10^-5 ^S which can be ascribed to the presence of Cu metallic particles. The introduction of Cu metallic particles to decorate CNTs can improve the wettability to CNTs with increasing numbers of vacancies in *d *orbital and reduce resistance of interface of CNT and ITO by enhancing the contact area so that the Cu-CNT assemblies' one tends to saturate slower [23]. The contact resistance can be described by [23]

(2) $$G=\frac{1}{R}=\underset{d}{\sum }\frac{N_{\text{d}}}{\left(r_{\text{c}}+r_{\text{t}}\right)}$$

where *G *is the contact conductivity, *d *the CNT diameter, *N*_d _the number of CNTs at a given diameter, *r*_c _the contact resistance of each CNT, and *r*_t _is the intrinsic resistance of an individual CNT. In Equation 2, *r*_c _can be given by [23]

(3) $$r_{\text{c}}=\frac{\kappa_{\text{B}}}{qA^{*}TS}\cdot \text{exp}\left(\frac{q\cdot \Delta \phi }{\kappa_{\text{B}}T}\right)$$

where *κ*_B _is the Boltzmann constant, *q *the electric charge, A*Richardson constant, *T *the absolute temperature, *S *the contact area, and Δ*ϕ *is the difference of barrier height. Since the Cu metal has similar work function to CNT, the Δ*ϕ *of Cu-CNT assemblies to ITO is almost the same as the bare-CNT's. Thus, the contact area determines the contact resistance of interface between CNT and ITO.

**Figure 7:**
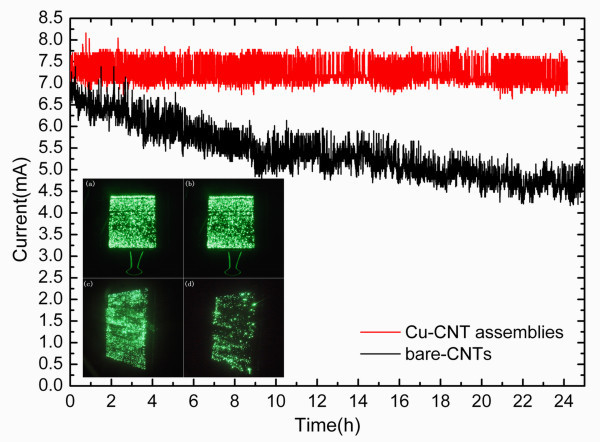
**Current-time curve of field emission stability and (inset) field emission luminescence images of green phosphor**: **(a) **Cu-CNT assembly cathode at beginning; **(b) **Cu-CNT assembly cathode after 24 h; **(c) **bare-CNTs cathode at beginning; and **(d) **bare-CNTs cathode after 24 h.

Figure 7 shows the curves of field emission current stability of both Cu-CNT assembly cathode and bare-CNTs cathode. It is in evidence that the introduction of Cu metallic particles to decorate the CNTs makes the field emission current tend to be undiminished over time despite of the existence of the field emission current fluctuation phenomena, and the current fluctuation range is uniform. In contrast with the bare-CNTs cathode, however, the field emission current is reduced gradually over time, and the fluctuation is uneven. It can be calculated that the emission current of the latter one decreases by 30% after continuous emission for 24 h. To explain the improvement brought by introduction of Cu metallic particles intuitively, the field emission luminescence images of green phosphor in Figure 7(inset) are compared. Insets a and b show the initial and after 24 h luminescence images using Cu-CNT assembly cathode while c and d using bare-CNTs cathode. Seen from insets a and c, both of the luminescence images have good uniformity at beginning. However, after keeping on field emission for 24 h, it is apparent to discover from insets b and d that the former still shows a better uniform image, in which the emitting area almost remains unchanged while the latter have many dark positions. The emergence of dark positions is because of the attenuation of field emission and the reason for attenuation is bad contact between CNTs and ITO which makes the contact resistance larger, accordingly, threshold field and thermal injury increase. The resulting instability because of decay is not good enough to be adopted in the display technology.

## Conclusion

In summary, the performance of introduction of Cu metallic particles to decorate CNTs field emitters by EPD method has been investigated. By means of SEM and EDS, we confirm that the EPD process is a simple and feasible way of fabricating Cu-CNT assembly cathode. The simulation of field distribution, field emission characteristic curve, and electron emission stability curve has been adopted to reveal the effect of Cu-CNT assemblies. The Cu-CNT assemblies have enhanced the electrical contact between CNTs and ITO electrode that the contact conductivity has greatly increased from 10^-5 ^to 4 × 10^-5 ^S and the turn-on field has been reduced from 3.4 to 2.2 V/μm. Meanwhile, the participation of Cu particles increases the field emitters. In addition, the field emission current tends to be undiminished over time. In contrast with the luminescence images, it is easy to find that the Cu-CNT assembly cathode indeed improves the field emission stability of CNT-FED. We expect that the introduction of Cu metallic particles to decorate the CNTs in this article will be an easy way to facilitate the improvement of emission stability of CNT-FED.

## Abbreviations

CNT-FED: carbon nanotube field emission display; CNTS: carbon nanotubes; Cu-CNT: copper metallic particle-carbon nanotube; EDS: energy dispersive X-ray spectroscopy; EPD: electrophoretic deposition; FED: field emission display; FN: Fowler-Nordheim. IPA: isopropyl alcohol; ITO: indium tin oxide; SEM: scanning electron microscope; XRD: X-ray diffraction.

## Competing interests

The authors declare that they have no competing interests.

## Authors' contributions

YC, DL and HS conceived of the study, and participated in its design and coordination. YC, ZL and XS carried out the experiments. HZ performed the statistical analysis and acquisition of data. YC drafted the manuscript. HJ and GM guided revised the manuscript. All authors read and approved the final manuscript.
